# Supplementary figures and images for: Dendrite intercalation between epidermal cells tunes nociceptor sensitivity to mechanical stimuli in Drosophila larvae
Source: PLoS Genet. 2024 Apr 25;20(4):e1011237. doi: 10.1371/journal.pgen.1011237 (PMC11075839; doi:10.1371/journal.pgen.1011237)

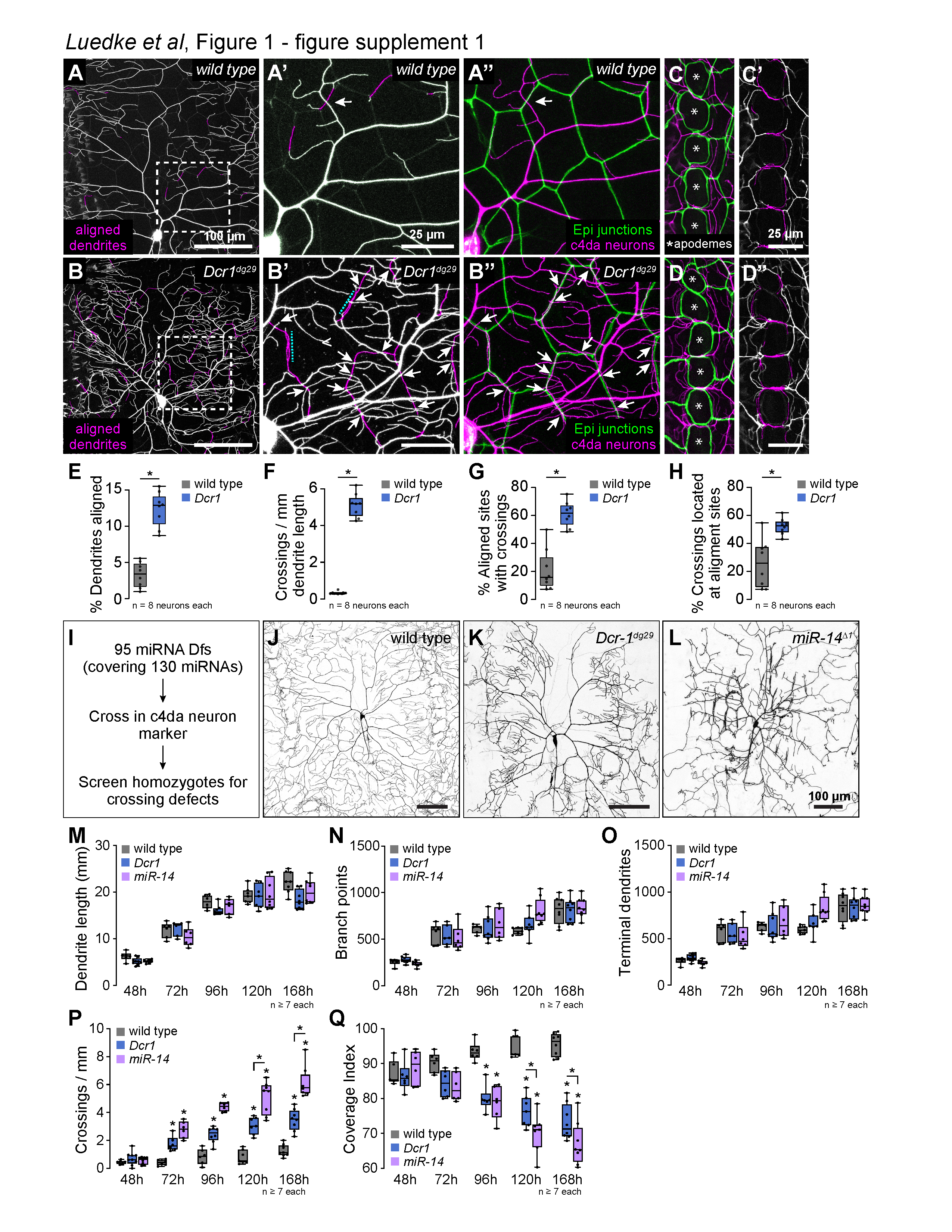

Supplement: S1 Fig — (A-D). Maximum intensity projections show C4da dendrites (ppk-CD4-tdTomato) of pre-EMS control (A, C) and Dcr1dg29 mutant larvae (B, D) at 120 h AEL that have been pseudocolored to show areas of dendrite alignment along epidermal junctions (Nrx-IVGFP). Insets (A’ and B’) show high magnification views of dendrite arbors, highlighting corresponding regions from control and Dcr1dg29 mutant larvae that contain junction-aligned dendrites. Arrows mark dendrite crossing events involving junction-aligned dendrites and dashed lines mark aligned dendrites that are bundled. (C, D) Maximum intensity projections show relative positions of C4da neurons and apodemes (asterisks). In both wild-type control (C) and (D) Dcr1dg29 mutant larvae, dendrites intercalate between and wrap around apodemes, but rarely innervate below apodemes. (E-H) Morphometric analysis of Dcr1dg29 C4da dendrite positioning defects. (E) Plot depicts the proportion of C4da dendrite arbors, excluding regions covering apodemes, that align along epidermal junctions. (F-H) Junction-aligned dendrites are frequently involved in homotypic dendrite crossing events. Plots depict (F) the frequency of homotypic dendrite crossing events within a C4da dendrite arbor (crossing number normalized to mm total dendrite length), (G) the proportion of junctional alignment sites within a C4da dendrite arbor that contain dendrite-dendrite crossing events, and (H) the proportion of homotypic dendrite crossing events within a C4da dendrite arbor that occur at sites of epidermal dendrite alignment. (I) Screen for miRNAs that control epidermal dendrite alignment. Deficiency alleles cover 130 miRNA genes, accounting for >99% of somatically expressed miRNAs [108]. (J-L) Representative images of C4da neurons from segment A3 at 96 h AEL in wild-type control (J), Dcr-1dg29 (K), and miR-14Δ1 mutant larvae (L). (M-Q) Morphometric analysis of C4da dendrites in Dcr-1dg29 and miR-14Δ1 mutant larvae. Mutations in Dcr-1dg29 and miR-14Δ1 have no si [file pgen.1011237.s001.tiff]

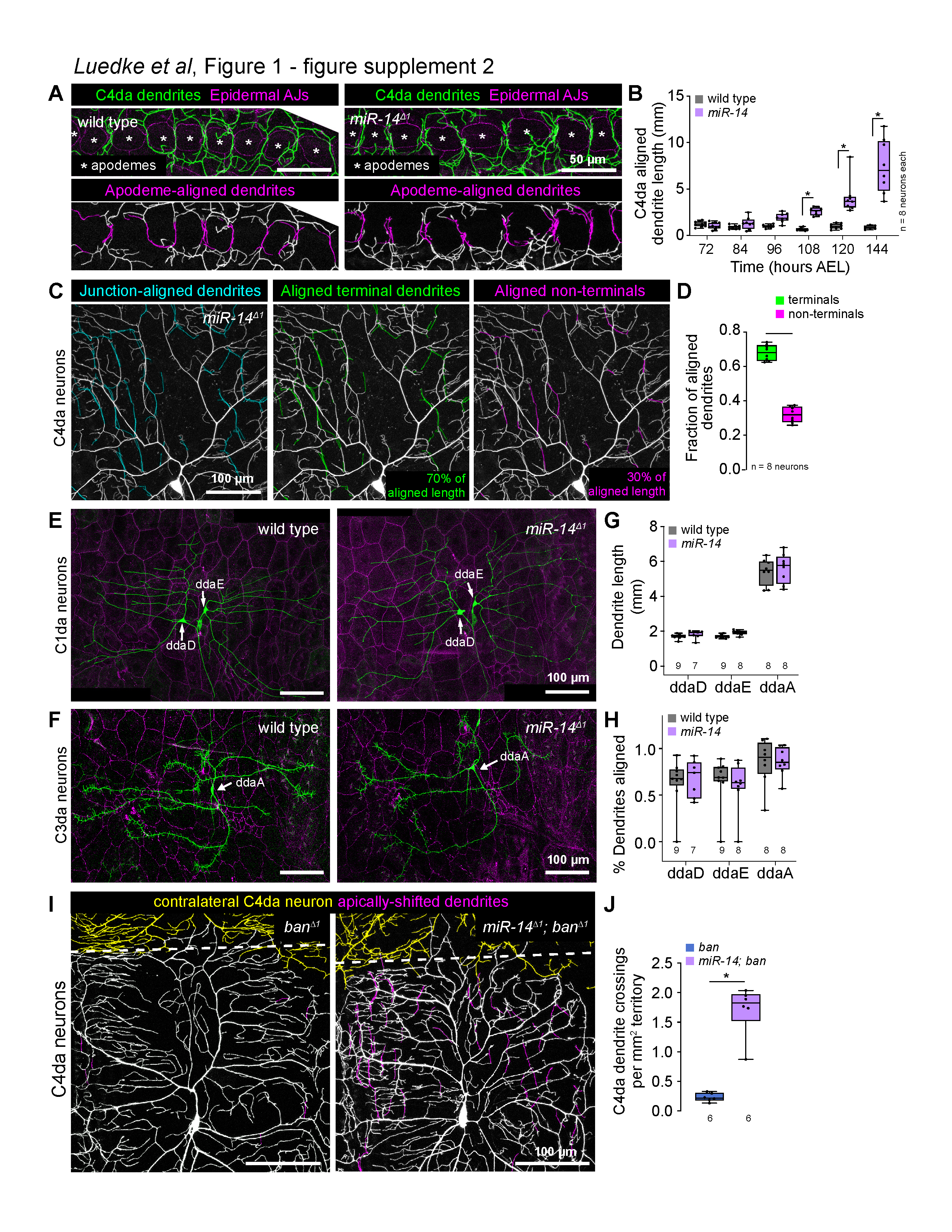

Supplement: S2 Fig — (A) Apodeme innervation is unaffected in miR-14 mutants. Maximum intensity projections show relative positions of C4da neurons and apodemes (asterisks) in wild-type control and miR-14 mutant larvae. In both backgrounds, dendrites intercalate between and wrap around apodemes (pseudocolored magenta), but rarely innervate below apodemes. (B) Plot depicts the total length of C4da dendrites aligned to non-apodeme epidermal junctions at the indicated developmental timepoints. *P<0.05, Kruskal-Wallis test with post-hoc Dunn’s test. (C-D) Distribution of epidermal junctional alignment in C4da dendrite arbors. (C) Maximum intensity projection of representative miR-14Δ1 mutant C4da neuron in which dendrites are pseudocolored cyan to indicate sites of epidermal junctional alignment, green to mark aligned terminal dendrites, and magenta to indicate aligned stretches that do not involve terminal dendrites. Dendritic alignment along epidermal junctions was identified by dendritic CD4-tdTomato (ppk-CD4-tdTomato) co-localization with the PIP2 marker PLCδ-PH-GFP. (D) Plot depicts the fraction of epidermal junction-aligned dendrites that involve terminal (green) and non-terminal (magenta) dendrites. *P<0.05, unpaired t-test with Welch’s correction. (E-H) miR-14 does not affect epidermal distribution of C1da or C3da dendrites. (E-F) Maximum intensity projections of wild-type control and miR-14 mutant larvae expressing shgRFP, which labels epidermal cell-cell junctions, and membrane-targeted CD4-tdGFP expressed in C1da neurons (E) or C3da neurons (F). Plots depict (G) total dendrite length and (H) proportion of dendrites that are aligned along epidermal junctions for the indicated genotype-cell type combinations. Comparing values from control and miR-14 mutant larvae using a Mann Whitney test revealed no significant differences. (I) Epidermal dendrite ensheathment and epidermal junctional dendrite alignment are regulated by distinct genetic pathways. Maximum intensity projections show [file pgen.1011237.s002.tiff]

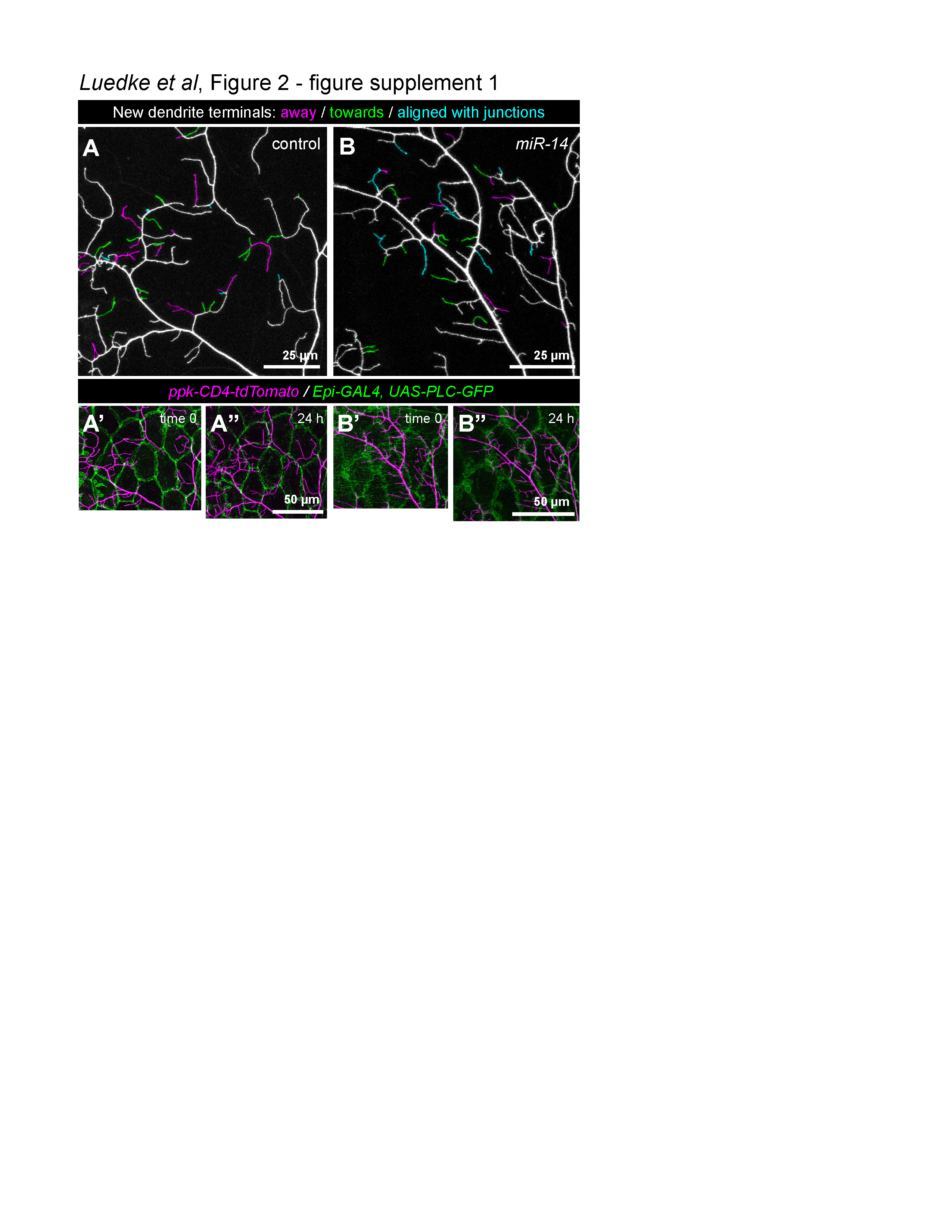

Supplement: S3 Fig — C4da neurons (ppk-CD4-tdTomato) were imaged over a 24 h time-lapse (96–120 h AEL) and the orientation of branch growth in relation to epidermal junctions (A58-GAL4, UAS-PLCδ-PH-GFP) was monitored for each new dendrite branch. Composite montages from representative for (A) wild-type control and (B) miR-14 mutant larvae show new dendrite branches pseudocolored according to following orientations: growth towards (green), away from (magenta) or aligned along epidermal junctions (cyan). Raw images for each genotype / time point combination are shown below composites. Experimental genotypes: wild type: w1118;; A58-GAL4, UAS-PLCδ-PH-GFP, ppk-CD4-tdTomato10A, miR-14: w1118; miR-14Δ1; A58-GAL4, UAS-PLCδ-PH-GFP, ppk-CD4-tdTomato10A. (TIFF) [file pgen.1011237.s003.tiff]

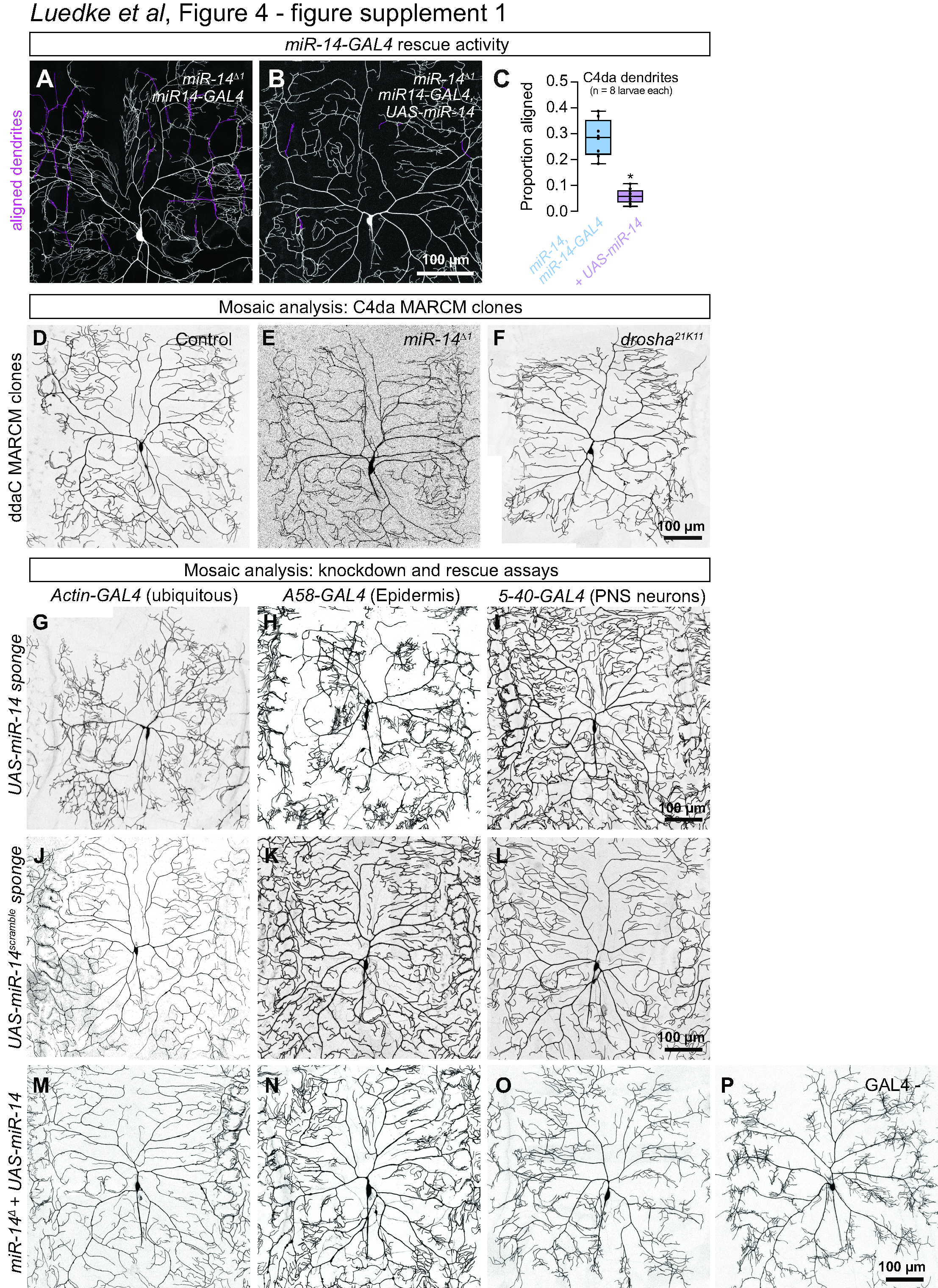

Supplement: S4 Fig — Composite images show morphology (white) and epidermal junctional alignment (magenta) of C4da dendrites in miR-14 mutant larvae that additionally express miR-14-GAL4 without (A) or with (B) UAS-miR-14. (C) UAS-miR-14 expression driven by miR-14-GAL4 rescues the miR-14 mutant junctional dendrite alignment defect. *P<0.05, unpaired t-test with Welch’s correction. (D-F) Related to Fig 4F. Maximum intensity projections show representative images of C4da neuron MARCM clones of the indicated genotypes. (G-L) Related to Fig 4G. Effects of miRNA sponge expression. C4da neurons from larvae expressing miR-14 sponge (G-I) or control sponge (J-L) with the indicated GAL4 driver. (M-O) Related to Fig 4H. miR-14 rescue assays. Maximum intensity projections show representative images of C4da neurons in miR-14 mutant larvae expressing UAS-miR-14 with the indicated GAL4 drivers. Experimental genotypes: (A-C) w1118; miR-14-GAL4, miR-14Δ1 / miR-14k10213, w1118; miR-14-GAL4, miR-14Δ1 / miR-14k10213; UAS-LUC-miR-14, (D-F) Genotypes listed in Fig 4F, (G-L) Genotypes listed in Fig 4G, (M-P) Genotypes listed in Fig 4H. (TIFF) [file pgen.1011237.s004.tiff]

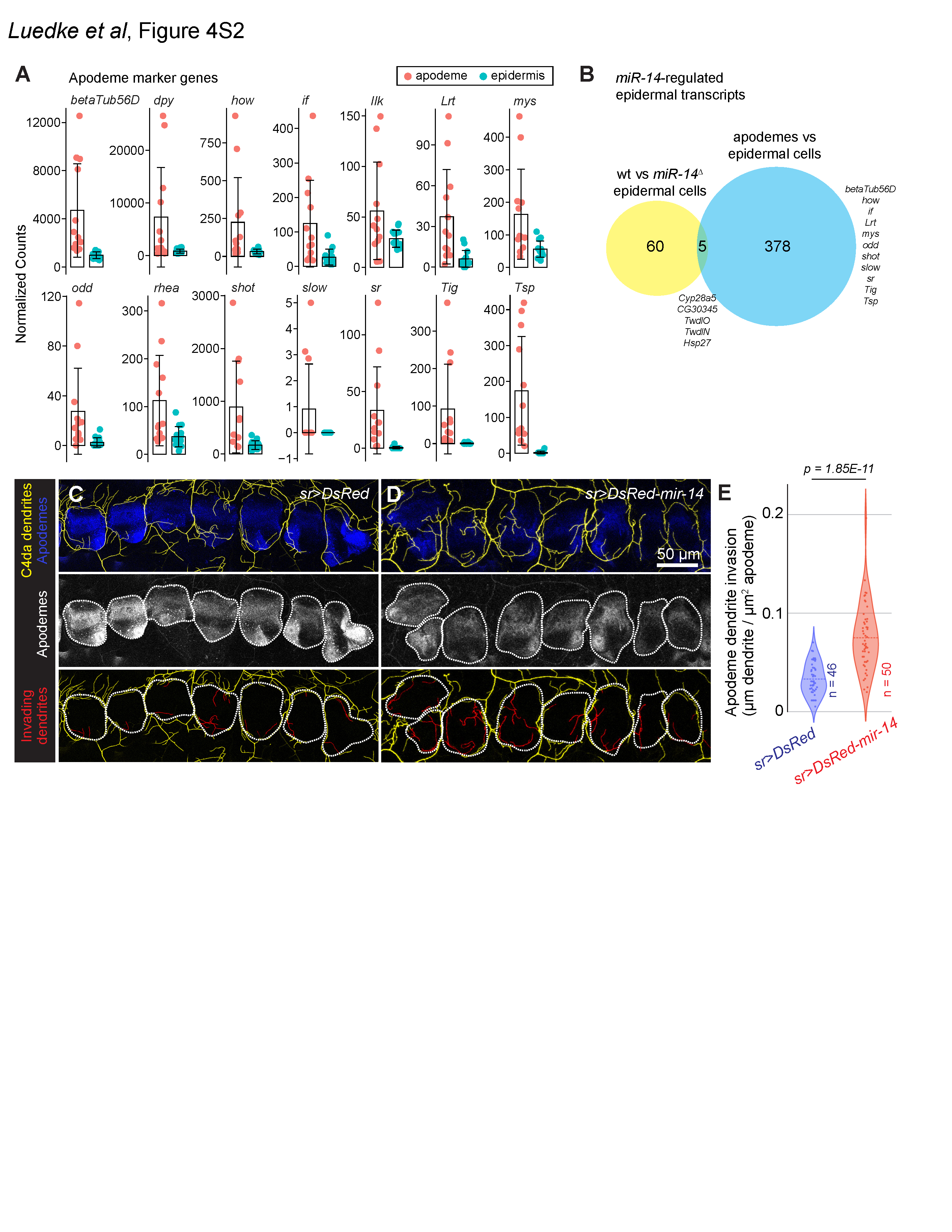

Supplement: S5 Fig — (A) Plots depict mRNA expression levels of apodeme marker genes in RNA-seq libraries generated from dissociated, manually picked apodemes (orange) and epidermal cells (teal). See also S1 Table for a complete list of genes differentially expressed between apodemes and epidermal cells. (B) miR-14 does not regulate expression of apodeme genes in epidermal cells. Venn diagram depicts overlap of genes differentially expressed between control and miR-14 mutant epidermal cells (yellow circle) and genes differentially expressed between apodemes and epidermal cells (blue circle). The intersection between the two datasets contains only five genes, none of which have known function in apodeme development or cell fate. (C-E) Ectopic expression of miR-14 in apodemes promotes denrite invasion into apodeme domains. (C-D) Representative maximum projection images from confocal stacks depict the distribution of C4da dendrites (ppk-CD4-tdGFP) over apodemes (sr-GAL4, UAS-DsRed) at segment boundaries in control larvae (C) or larvae overexpressing miR-14 selectively in apodemes (D). Apodeme boundaries are marked by hatched lines. (E) Violin plot depicts the density of dendrite invasion on the basal surface of apodemes. Each point represents a measurement of dendrite coverage from an individual apodeme. P = 1.85 x 10^−11^, unpaired T-test with Welch’s correction. Experimental genotypes: (A-B) Epidermal cells: w1118; R38F11-GAL4 / UAS-2x-EGFP, miR-14 epidermal cells: w1118; miR-14Δ1 / miR-14k10213; R38F11-GAL4 / UAS-2x-EGFP, Apodemes: w1118; UAS-2x-EGFP/+; sr-GAL4 / +, (C-E) sr>DsRed: ppk-CD4-tdGFP / +; sr-GAL4 / UAS-DsRed, sr>DsRed-miR-14: ppk-CD4-tdGFP / +; sr-GAL4 / UAS-DsRed-miR-14. (TIFF) [file pgen.1011237.s005.tiff]

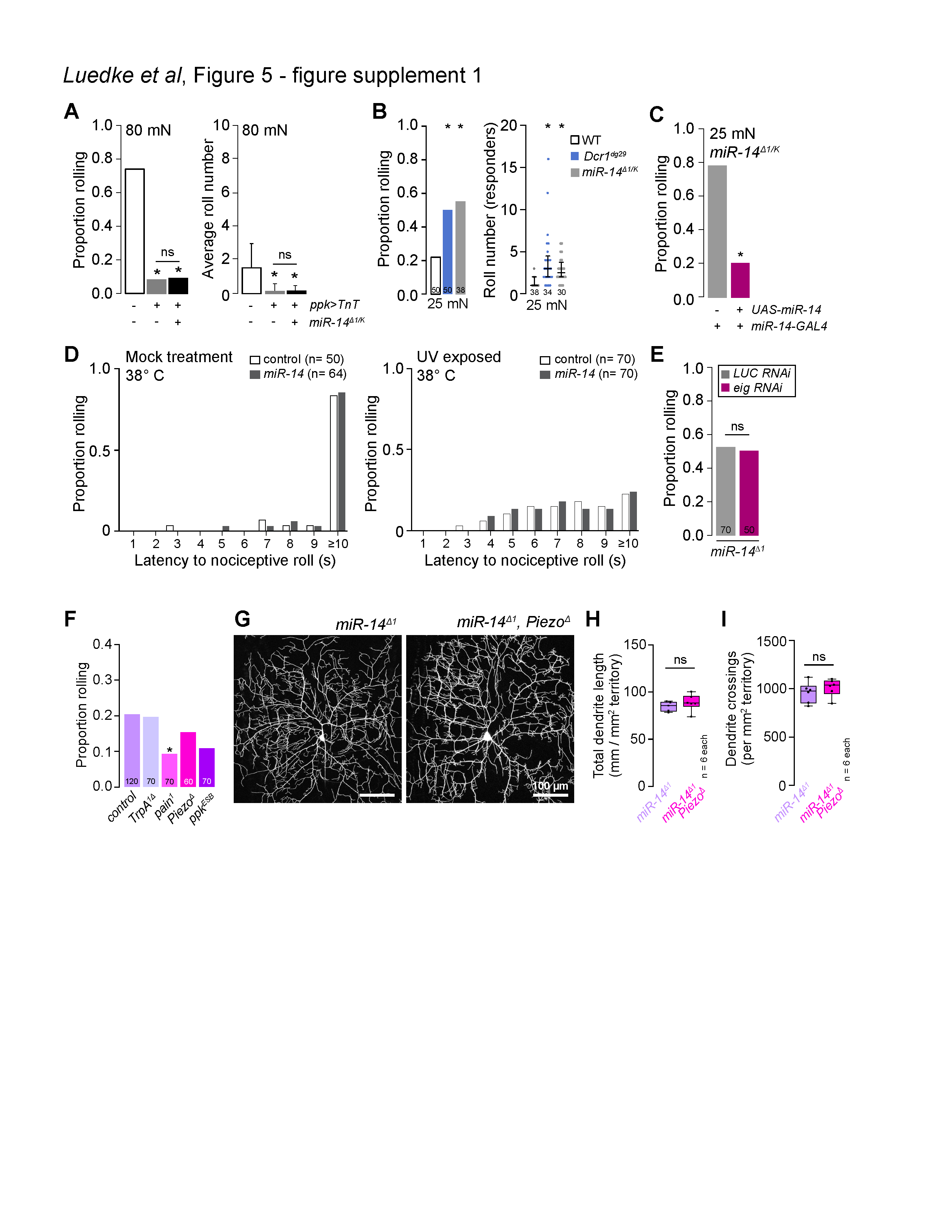

Supplement: S6 Fig — (A) C4da neuron activity is required for miR-14 mutant hypersensitivity to mechanical stimuli. Expressing UAS-Kir2.1 in nociceptive C4da neurons suppresses miR-14 mutant mechanical hypersensitivity. Plots depict the proportion rolling (left) and mean roll number (right) of larvae of the indicated genotype to 80 mN von Frey stimulation. *P<0.05, Fisher’s exact test with a BH correction (proportion rolling) or Kruskal-Wallis test followed by Dunn’s multiple comparisons test (roll number). (B) Dcr1 mutants and an additional miR-14 allelic combination exhibit enhanced nocifensive behavior responses. Plots depict proportion of larvae that exhibit nocifensive rolling and mean number of nocifensive rolls in response to von Frey fiber stimulation of the indicated intensities. *P<0.05, Fisher’s exact test with a BH correction (proportion rolling) or Kruskal-Wallis test followed by Dunn’s multiple comparisons test (roll number). (C) UAS-miR-14 expression driven by miR-14-GAL4 rescues the miR-14 mutant nociception sensitization phenotype. *P<0.05, Fisher’s exact test. (D) miR-14 is dispensible for UV-induced thermal allodynia. Plots depict latency values (seconds) to the first nociceptive roll in response to 38°C stimuli for control and miR-14 mutant larvae 24 h following mock treatment or UV irradiation. Chi-square test revealed no significant difference between the two genotypes. (E) Epidermal expression of the TNF ligand Eiger is dispensable for nociceptive sensitization in miR-14 mutant larvae. Plot depicts nociceptive rolling responses to 25 mN stimulus of miR-14 mutant larvae expressing Luciferase-RNAi or eiger-RNAi in epidermal cells. NS, no signicant difference, Fisher’s exact test. (F) Piezo is largely dispensable for nociceptive rolling responses induced by a 25mN stimulus. Plots depict nocifensive rolling responses to 25 mN von Frey fiber stimulation of control or channel mutant larvae. *P < 0.05, Kruskal-Wallis test followed by a Dunn’s multiple comparisons test. ( [file pgen.1011237.s006.tiff]

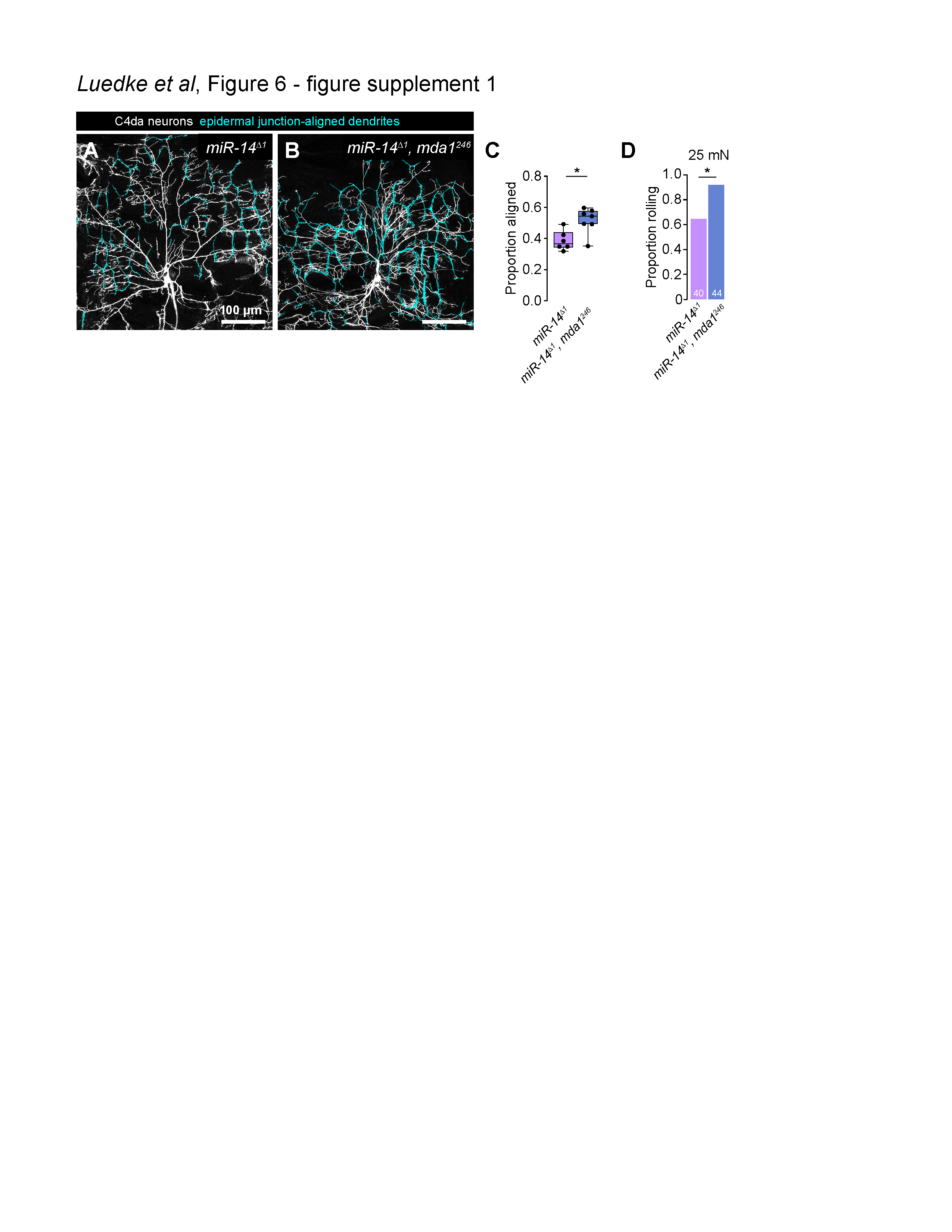

Supplement: S7 Fig — Representative composite images of C4da dendrites pseudocolored cyan at sites of epidermal junctional alignment are shown for (A) miR-14Δ1 mutant and (B) miR-14Δ1, mda1246 double mutant larvae. Sites of dendrite alignment to epidermal junction were identified as sites of colocalization between GFP (ppk-CD4-tdGFP) and anti-cora immunoreactivity. (C) Plot depicts the proportion of dendrite arbors aligned along epidermal junctions in larvae of the indicated genotypes. *P<0.05, unpaired t-test with Welch’s correction. (D) Plot depicts nociceptive rolling responses of larvae of the indicated genotypes to 25 mN von Frey stimulation. *P<0.05, Fisher’s exact test. Experimental genotypes: (A-D) w1118; miR-14Δ1, ppk-CD4-tdGFP1b, w1118; miR-14Δ1, mda1246, ppk-CD4-tdGFP1b. (TIFF) [file pgen.1011237.s007.tiff]

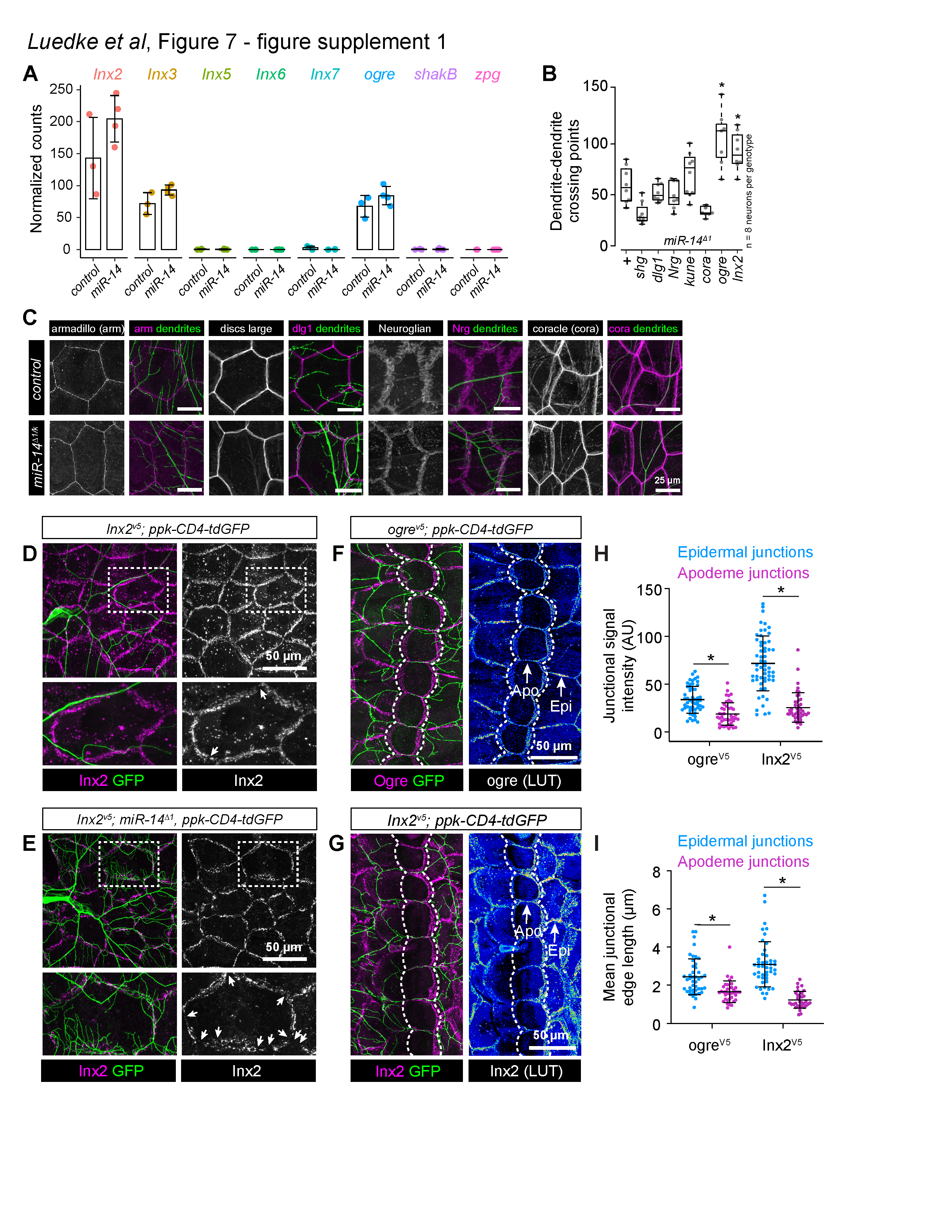

Supplement: S8 Fig — (A) Epidermal expression of Inx genes. Plot depicts mean and standard deviaion for mRNA levels (normalized counts) for the indicated Innexin genes from RNA-seq analysis of epidermal cells. Points indicate expression values from independent biological replicates. (B) Morphometric analysis of C4da dendrites in larvae heterozygous for miR-14Δ1 and loss-of-function mutations in the indicated epidermal junction genes showing the mean number of dendrite-dendrite crossing events per neuron. *P<0.05 compared to miR-14 heterozygous controls, ANOVA followed by post-hoc Dunnett’s test. (C) Expression and distribution of AJ and SJ markers in control and miR-14 mutant larvae. Representative images show expression of the AJ markers armadillo and discs large (C, D), and the septate junction markers coracle (E, F) and neuroglian (G, H) in individual epidermal cells of control or miR-14 mutant larvae expressing ppk-CD4-tdGFP to label C4da dendrites. miR-14 mutation did not cause substantial alterations in level or distribution of these markers. (D-E) miR-14 regulates epidermal Inx2 distribution. Maximum intensity projections show distribution of Inx2 in the epidermis of wild type control (D) and miR-14 mutant larvae (E). As with ogre immunoreactivity (Fig 7), miR-14 mutation caused irregularities in the belt of Inx2 immunoreactivity including frequent discontinuities (arrows). (F-I) GJ proteins are differentially expressed at apodemes and other epidermal cells. Maximum intensity projections (top) show C4da dendrites in green and the GJ proteins ogre (F) and Inx2 (G) in magenta, and lookup tables depict Inx intensity. Hatched lines outline apodeme boundaries, and cell-cell interfaces between apodemes (Apo) and epidermal cells (Epi) are indicated with arrows. Plots depict (H) ogre and Inx2 intensity and (I) the cross-sectional width of ogre and Inx2 immunoreactivity at cell-cell interfaces of apodemes and other epidermal cells. *P<0.05, Kruskal-Wallis test followed by Dunn’s multiple [file pgen.1011237.s008.tiff]

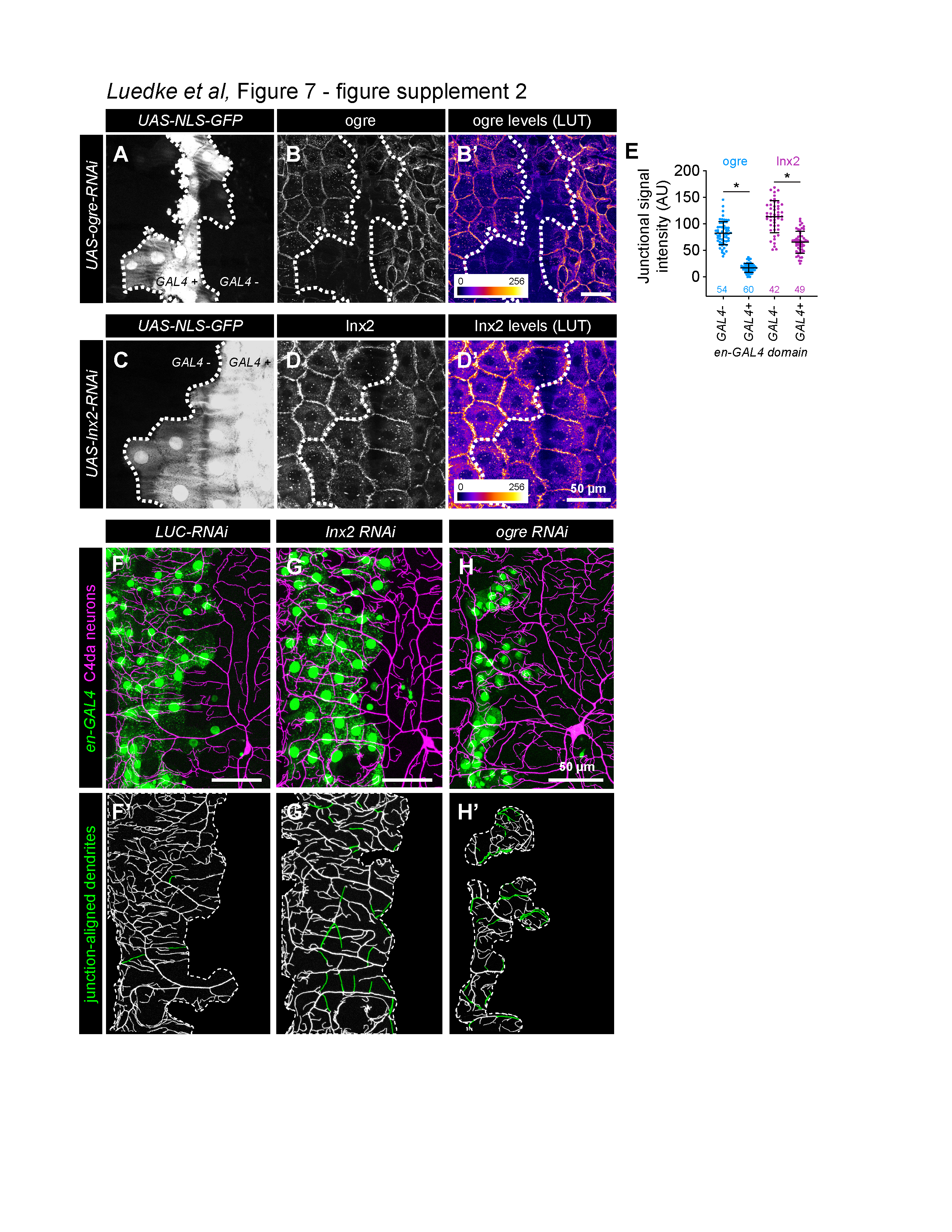

Supplement: S9 Fig — Representative images depict effects of UAS-ogre-RNAi (A-B) or UAS-Inx2-RNAi (C-D) expression on Inx protein levels. (A, C) Maximum intensity projections show anti-GFP immunoreactivity to label the en-GAL4 expression domain and (B, D) anti-V5 immunoreactivity to label V5-tagged endogenous ogre (B) or Inx2 (D), (B’, D’) Inx protein levels pseudocolored according to a lookup table. White hatched lines demarcate boundaries of en-GAL4 expression domains. (E) Plot depicts ogre and Inx2 intensity at epidermal junctions outside (GAL4-) or inside (GAL4+) the en-GAL4, UAS-RNAi expression domain. *P<0.05, ANOVA with post-hoc Sidak’s test. (F-H) Double labeling of C4da dendrites (ppk-CD4-tdGFP) and epidermal cells (en-GAL4, UAS-NLS-GFP) additionally expressing (F) UAS-LUC-RNAi (control), (G) UAS-Inx2-RNAi, or (H) UAS-ogre-RNAi. Traces show dendrite arborization and epidermal junction-aligned dendrites (pseudocolored green) within the en-GAL4 expression domain. Experimental genotypes: (A-E) ogre RNAi: ogreV5; en2.4-GAL4, UAS-RedStinger, ppk-CD4-tdGFP1b/+; UAS-ogreRNAi/+, Inx2 RNAi: Inx2V5; en2.4-GAL4, UAS-RedStinger, ppk-CD4-tdGFP1b/+; UAS-Inx2-RNAi/+, (F-H) LUC RNAi: w1118; en2.4-GAL4, UAS-RedStinger, ppk-CD4-tdGFP1b/+; UAS-LUC-RNAi/+, ogre RNAi: w1118; en2.4-GAL4, UAS-RedStinger, ppk-CD4-tdGFP1b/+; UAS-ogreRNAi/+, Inx2 RNAi: w1118; en2.4-GAL4, UAS-RedStinger, ppk-CD4-tdGFP1b/+; UAS-Inx2-RNAi/+ (TIFF) [file pgen.1011237.s009.tiff]
