# Supplementary material for: Dendrite intercalation between epidermal cells tunes nociceptor sensitivity to mechanical stimuli in Drosophila larvae
Source: PLoS Genet. 2024 Apr 25;20(4):e1011237. doi: 10.1371/journal.pgen.1011237 (PMC11075839; doi:10.1371/journal.pgen.1011237)
Supplement: S2 Table — Each allele is listed according to its use in this study along with the corresponding RRID identifier. (PDF) [file pgen.1011237.s011.pdf]

Table S2. Alleles used in this study

Allele	Uses / Features	Identifier (RRID)
5-40-GAL4	GAL4 driver (PNS neurons)	Flybase_FBal0221791
98b-GAL4	GAL4 driver (C1da neurons)	Flybase_FBal0305321
Act5C-GAL4	GAL4 driver (ubiquitous)	BDSC_3954
A58-GAL4	GAL4 driver (epidermis)	Flybase_FBal0181674
en2.4-GAL4	GAL4 driver (epidermis)	BDSC_30564
miR-14-GAL4	GAL4 driver (*miR-14* expression domain)	This study
NompC-GAL4	GAL4 driver (C3da neurons)	BDSC_36361
ppk-GAL4	GAL4 driver (C4da neurons)	BDSC_32079
R38F11-GAL4	GAL4 driver (epidermis)	BDSC_50014
sr-GAL4	GAL4 driver (apodemes)	BDSC_26663
ppk-LexA	LexA driver (C4da neurons)	Flybase_FBal0336342
UAS-mCherry.scramble.sponge	Knockdown (control miRNA sponge)	BDSC_61501
UAS-mCherry.miR-14.sponge	Knockdown (*miR-14* sponge)	BDSC_61382
$UAS\text{-}eiger^{IR}$	Knockdown (*eiger* RNAi line)	BDSC_58993
UAS-Inx2RNAi	Knockdown (*Inx2* RNAi line)	BDSC_42645
UAS-LUC	Knockdown (*Luciferase* RNAi line)	BDSC_31603
UAS-ogreRNAi	Knockdown (*ogre* RNAi line)	BDSC_44048
FRT42D	MARCM reagent	BDSC_1802
SOP-FLP	MARCM reagent	Flybase_FBal0278148
Tub-GAL80	MARCM reagent	BDSC_9917
$ban^{\Delta 1}$	Mutant allele	BDSC_58878
$cora^{5}$	Mutant allele	BDSC_52233
$Dcr1^{mn29}$	Mutant allele	This study
$dlg1^{A}$	Mutant allele	BDSC_57086
$Drosha^{21K11}$	Mutant allele	Flybase_FBal0268190
$inx2^{G0118}$	Mutant allele	BDSC_11826
$kune^{C309}$	Mutant allele	BDSC_16333
$mdi1^{242}$	Mutant allele	This study
$mdi2^{246}$	Mutant allele	This study
$mdi3^{442}$	Mutant allele	This study
$mdi4^{51}$	Mutant allele	This study
$miR\text{-}14^{\Delta 1}$	Mutant allele	BDSC_33067
$miR\text{-}14^{k10213}$	Mutant allele	BDSC_10982
nan^{GAL4}	Mutant allele	BDSC_68205
$NompC^{1}$	Mutant allele	BDSC_42260
$NompC^{3}$	Mutant allele	BDSC_42258
Nrg^{14}	Mutant allele	BDSC_5708
$ogre^{1}$	Mutant allele	Flybase_FBal0013231
$pain^{1}$	Mutant allele	BDSC_27895
$piezo^{KO}$	Mutant allele	BDSC_58770
ppk^{ESB}	Mutant allele	BDSC_79622
shg^{2}	Mutant allele	BDSC_3085
Tig^{A1}	Mutant allele	BDSC_8795
Tig^{x}	Mutant allele	BDSC_8796
$TrpA1^{1}$	Mutant allele	BDSC_26263
UAS-dsRed-miR-14	Overexpression/rescue construct	BDSC_59868
UAS-if	Overexpression/rescue construct	Flybase_FBal0062798
UAS-Inx2.Sb	Overexpression/rescue construct	Flybase_FBal0338197
UAS-LUC-miR-14.T	Overexpression/rescue construct	BDSC_41178
UAS-mew	Overexpression/rescue construct	Flybase_FBal0062567
UAS-ogre.S	Overexpression/rescue construct	Flybase_FBal0338192
UAS-TNT	Overexpression/rescue construct	BDSC_28997
$Ilk^{ZCL3111}$	Reporter (muscle adhesion sites; GFP-tagged Ilk)	BDSC_6831

inx2^{V5}	Reporter (V5-tagged Inx2)	Flybase_FBal0345156
Mhc::mCherry	Reporter (muscle)	Flybase_FBal0358776
ogre^{V5}	Reporter (V5-tagged Ogre)	Flybase_FBal0345155
Nrg167GFP	Reporter (epidermal septate junctions)	BDSC_6844
Nrx-IV-GFP	Reporter (epidermal septate junctions)	BDSC_50798
ppk-CD4-tdGFP^{1b}	Reporter (C4da neurons)	BDSC_35842
ppk-CD4-tdTomato^{10A}	Reporter (C4da neurons)	BDSC_35845
rhea$^{MI00296\text{-}mCh.0}$	Reporter (Apodemes; Cherry-tagged Rhea)	BDSC_39648
shg^{mCherry}	Reporter (epidermal adherens junctions)	BDSC_59014
trol-GFP	Reporter (epidermal BM)	Flybase_FBal0243610
Tub-GFP	Reporter (control miRNA sensor)	FBtp0017439
Tub-GFP.mir-14	Reporter (*miR-14* sensor)	FBtp0056974
UAS-2xEGFP	Reporter (cytosolic EGFP)	BDSC_60293
UAS-CD4-tdGFP	Reporter (membrane-targeted GFP)	BDSC_35839
UAS-dsRed	Reporter (RFP)	BDSC_6282
UAS-GCaMP6s	Reporter (calcium imaging)	BDSC_42749
AOP-GCaMP6s	Reporter (calcium imaging)	BDSC_44273
UAS-Gerry	Reporter (ratiometric calcium imaging)	BDSC_80141
UAS-mCD8-GFP	Reporter (membrane-targeted GFP)	BDSC_5137
UAS-NLS-GFP	Reporter (nuclear-targeted GFP)	BDSC_4776
UAS-PLC$^{\delta}$*-PH-GFP*	Reporter (PIP2 reporter)	BDSC_39693
UAS-RedStinger^{4}	Reporter (NLS-RFP)	BDSC_8546
UAS-tdTomato	Reporter (RFP)	BDSC_36328
