# Supplementary material for: Dendrite intercalation between epidermal cells tunes nociceptor sensitivity to mechanical stimuli in Drosophila larvae
Source: PLoS Genet. 2024 Apr 25;20(4):e1011237. doi: 10.1371/journal.pgen.1011237 (PMC11075839; doi:10.1371/journal.pgen.1011237)
Supplement: Attachment — Submitted filename: Parish Plos Genetics.pdf [file pgen.1011237.s013.pdf]

Review of **PGENETICS-D-23-01043**

Luedke et al present a fascinating study of phenotypes that are observed in Drosophila mutants for Dicer-1 and mir-14. Mutations in both genes are found to affect the dendritic arborization of nociceptive neurons in the Drosophila larvae. The sensory dendrites of mutants for these genes are found to penetrate through the cell-cell boundaries of overlying epidermal cells, a pattern that is not normally seen in wild type larvae. Multiple and convincing lines of evidence suggest that mir-14 is required in epidermal cells to prevent the dendrites from penetrating at these boundaries. Furthermore, it is found that the mir-14 mutants have hypersensitive mechanical nociception and this phenotype is correlated with the boundary penetration of the dendrites as it can be suppressed by overexpression of integrin genes. Finally, it is found that epidermal gap junctions are disrupted by mir-14 mutations and manipulating gap junction gene expression in epidermal cells phenocopies the dendrite and behavioral effects. Overall, this is a very exciting study that would be of great interest to the readers of PLoS Genetics but there are some questions that have been raised by the findings and their interpretation that should be addressed.

Major Points:

1.) The authors dedicate a lot of discussion to the distinction between apodemes (aka muscle attachment sites) and epidermal cells. It is nice that this paper describes the observation that the dendrites of nociceptive neurons pass between the muscles at the site of their attachment as this is something that is well-known to those of us that are interested in these neurons, but this has not been well-described in the literature. However, throughout the manuscript the reader is given the impression that there is something special about the apodemes themselves that causes the dendrites to pass around their boundaries. There is no discussion whatsoever of the fact that the muscles, which attach to these apodemes, present a physical barrier that the dendrites simply cannot pass through. While it is possible that there are cues that guide the dendrites along the apodemal boundaries, it seems more plausible that the muscles themselves are the factor that block the dendrites from entering into this territory. This latter idea is actually supported by the authors own exciting data which are presented in figure 8L! Tiggrin mutants, which are defective in muscle attachment, show dendrite penetration into the epidermal territory. Can the authors revise the manuscript to more clearly present the tissue level structure at the apodemes? There’s a muscle in the way.

2.) Although the Tiggrin phenotype is suggestive that muscles pose a physical barrier to dendrites, it is also possible that the edges of apodemes provide some additional guidance cues to facilitate the dendrites passing around their edges. And it seems like the authors have found that low expression of gap junctions could be the relevant cues. Does mir-14 expression in epidermal cells suppress the apodeme fate? It seems like it would be interesting/important to investigate whether or not epidermal cells in mir-14 mutants show expression of other apodemal markers, there are good markers out there to look at this quickly and with little effort.

3.) While it seems very clear that the mir-14 mutants show hypersensitive mechanical nociception, it is less clear that the penetration of dendrites into epidermal junctions has anything to do with wild type nociception. This statement does is not meant to detract from the interesting phenotype, but it is not clear that wild type neurons penetrate at these boundaries at all (besides at apodemes). Is the fraction of dendrites aligned with epidermal cells in Figure 1K significantly above the alignment that would be expected by chance? If not, the manuscript should be revised to state that normal nociception may or may not be influenced by these specific dendrite epidermal interactions.

4.) The data presented in figure 8 G-J are completely unconvincing. The signals in panel I do not resemble genuine GCaMP responses. The description of the methodology employed is not adequate for these data to be evaluated by a reviewer. These data add very little information to the manuscript, and the easiest thing for the authors to do would be to just remove the data. It is extremely challenging to rigorously analyze calcium responses in a moving preparation and these experiments are not credible as currently presented.

5.) It's interesting that mir-14 mutants don't cause thermal hyperalgesia and the effect is specific to mechanical, recommend moving Figure 5D supplement 1D to main figure.

6.) Congratulations to the authors for a beautiful and fascinating study.
