# Supplementary material for: Dendrite intercalation between epidermal cells tunes nociceptor sensitivity to mechanical stimuli in Drosophila larvae
Source: PLoS Genet. 2024 Apr 25;20(4):e1011237. doi: 10.1371/journal.pgen.1011237 (PMC11075839; doi:10.1371/journal.pgen.1011237)
Supplement: Attachment — Submitted filename: Luedke et al 2023 revision cover letter.pdf [file pgen.1011237.s014.pdf]

DEPARTMENT OF BIOLOGY
UNIVERSITY of WASHINGTON

December 15, 2023

RE: Revised submission of PGENETICS-D-23-01043

Dear Dr. Yu and editorial team,

We wanted to thank you and the Reviewers for considering our manuscript "*Dendrite intercalation between epidermal cells tunes nociceptor sensitivity to mechanical stimuli in Drosophila larvae*" for publication in *PLoS Genetics*. We were pleased that each of the Reviewers recognized significant strengths in our study, and additionally appreciate their efforts to identify opportunities for improving the study. We revised our manuscript according to the Reviewers' suggestions, incorporating several new results that substantially improve the study including:

- New imaging studies to clarify the anatomical relationship between nociceptive C4da dendrites, muscles, and apodemes
- New RNA-seq studies to evaluate expression differences between apodemes and other epidermal cells
- New RNA-seq and imaging studies to examine putative functions for *miR-14* in repressing apodeme cell fate
- New analyses to further explore effects of various treatments (Integrin overexpression, Tiggrin mutation) on dendrite morphology
- New quantitative analysis of epidermal gap junction defects
- New behavioral studies of sensory channel mutants

In addition to these experimental additions, we have made changes to the text and several figures which improve the overall readability of the manuscript. We are therefore please to present this revised manuscript and hope that you find it suitable for publication in *PLoS Genetics*.

Please find our detailed response to Reviewers' comments appended below.

Sincerely,

Jay Parrish, Ph.D.
Professor of Biology

Jay Parrish, PhD / Professor of Biology
Box 351800 / Life Sciences Building / Seattle, WA 98195-1800
206.685.1203 / jzp2@uw.edu / theparrishlab.com

Point-by-point response to Reviewer's comments (PGENETICS-D-23-01043)

Reviewer 1

1. *In the manuscript, the authors only studied Ogre and Inx2 to relate miR14 function. From target scan database in mirbase, Inx3 is predicted as the direct target of miR-14. Have the authors checked whether the expression level of Inx3 is increased in miR-14 mutant? How about the genetic interaction with miR-14? If the inx3 is upregulated in miR-14 mutants, the increased levels could also interfere gap junction integrity and lead to dendrite alignment increase.*

The Reviewer raises an interesting question; we omitted discussion of *Inx3* because (1) we have no evidence that it is a direct target of *miR-14* (discussed below; new results incorporated into the revision to address this point), (2) there is limited evidence of it functioning independently of Inx1 or Inx2 (as discussed in the manuscript), and (3) available alleles to study *Inx3* function or distribution are limited. It would be possible to generate new alleles, but we determined that such studies would not substantially increase the impact of the study.

As a first approach towards evaluating whether *Inx3* (or other GJ genes) were direct targets of *miR-14*, we used RNA-seq to compare gene expression profiles of control and *miR-14* mutant epidermal cells. Although we identified 65 differentially expressed genes (with an FDR of 0.05; ~85 genes with an FDR of 0.1), neither *Inx3* nor any other GJ gene was differentially expressed in *miR-14* mutants. We incorporated these results into Figure 7S1 (Fig. 7S1B). We additionally modified the text to reflect the fact that *Inx3* was identified as a predicted *miR-14* target.

2. Fig. 1 and legend need revision:
(i) Fig 1A, histoblasts (indicated in Fig 1B) at left bottom are masked!
We repositioned the labels to unmask the histoblasts.

(ii) Fig 1E' shows aligned dendrite segments that are supposed larger than 5 um (described in Image analysis). Some small ones are obviously smaller than 5um!
We appreciate the reviewer for catching this error; the value should have read 2 uM, which is the same value we used in our analysis shown in Fig. 3.

(iii) Legend does not match Figures, e.g. (H) shows aligned length is described as ensheathed, and also (I-K) are not either described or mis-described.
We thank the reviewer for catching this oversight; it was a holdover from a prior version of the figure and has been updated.

(iv) in Fig1S2C, the aligned non-terminals image (right panel) is not properly presented, as the terminal portions are cropped. This is misleading to readers when viewing the image as the magenta colored portions appear to be terminal!
We thank the Reviewer for catching this masking error. We updated the panel to include a version of the image with the aligned terminal dendrites unmasked (visible).

3. ***Are there contributions to dendrite intercalation from epidermal attachment to muscle cells?***
This is an intriguing question. Although a detailed analysis of muscle-derived cues merits its own standalone study, we incorporated new imaging results to better depict the anatomical relationship between dendrites and muscle adhesion sites. Specifically, we labeled muscles with a *MHC-Cherry* reporter transgene, muscle adhesion sites with an *ILK-GFP* exon trap allele, and C4da neurons with the *ppk-CD4-tdTomato* reporter. As shown in Fig 8G, C4da dendrites are largely confined to regions between muscle adhesion sites, suggesting that dendrite intercalation between apodemes may be a consequence of physical occlusion from muscle attachments. Taken together with our studies of *Tiggrin* mutations, which compromise muscle attachments, our studies indeed suggest that muscle attachments contribute to dendrite orientation at apodemes. Furthermore, phenotypes of *Tiggrin* mutants (in which dendrites aggregate over apodemes) suggest that apodemes may contain attractive cues that orient dendrite growth; we briefly address this in the discussion, but detailed study of apodeme-derived signals (and muscle-derived signals) merits its own standalone study.

4. Fig 3E, the cytosolic RFP appears to be very punctated?
We thank the Reviewer for drawing our attention to this point. The cytosolic RFP does indeed aggregate in some specimens, though we have not observed a systematic difference based on genotype. The degree of aggregation in the prior version of the figure was not representative of what we observe in properly fed/staged larvae, so we replaced the image with a more representative specimen (Fig. 3E).

5. ***The conclusion in lines 369-370 is not well supported. The effect in suppressing miR14 mutants in both dendrite alignment to cell junctions and nociefensive rolling responses could be also due to dendrite attachment to basement membranes and/or enshealthment. (Since Integrins overexpression had no effect on dendrite intercalation in wild type background (Fig 6E), but suppressed nociefensive rolling responses (80mN in Fig 6F) in wild type larvae. I suggest the conclusion should be modified, and the figure can be moved to Supplement.***
We agree with a portion of this interpretation but disagree on a critical point which, we believe, supports our conclusion. Indeed, we found that integrin overexpression attenuates responses to 80mN stimuli, and this is consistent with our prior results that blocking ensheathment (a consequence of integrin overexpression) similarly attenuates responses to 80mN stimuli. However, we observed <u>no effect</u> of integrin expression on larval responses to 20mN stimuli, and this is the relevant control.

In the revision we explored one possible alternative explanation - that integrin overexpression influences dendrite branching in *miR-14* mutants. However, we found that integrin overexpression had no significant effect on the overall dendrite length or terminal dendrite number in *miR-14* mutant C4da neurons. We incorporated these new results into Figure 6 and we have more carefully worded the results section and our discussion of the result to clarify the distinction between larval responses to these two force regimes.

6. ***The inclusion of mda mutants does not provide much support as the information provided is limited. I suggest to skip the data in this manuscript.***
We include the study of *mda1* to make one important point, namely that that further increases in intercalation are associated with further enhancement of mechanical nociceptive sensitivity. That this mutant is, aside from the EMS-induced mutations, isogenic with the *miR-14* allele we use for comparisons makes the results even more meaningful. As described below, we attempted to identify the lesion responsible for the mutant phenotype but have so far been unsuccessful. To clarify our takeaway from studies of this mutant we reworded the text as follows, replacing "connection" with the more appropriate "correlation":

> From this screen we identified one enhancer mutation (*mda-1*, *modifier of miR-14 dendrite alignment*) that significantly increased the extent of epidermal dendrite alignment in *miR-14* mutants (Fig. 6S1A-6S1C) as well as nociceptive sensitivity (Fig. 6S1D), further underscoring the correlation between epidermal dendrite intercalation and larval sensitivity to noxious mechanical inputs.

7. ***The study of gap junction proteins shows transheterozygous genetic interaction, regulation of protein levels, and the suppression of miR14 mutant by overexpression indicate a major functional regulation of miR-14, and the involvement of gap junction protein in dendrite intercalation. These data are important, and I wonder why these data are not shown as main figures*.**
We agree that the results support a major role for *miR-14* in regulation of GJ proteins. The data that the Reviewer referred to is indeed in the main figures (Fig 7), and in the revision we added new analyses that quantitatively assess effects of *miR-14* mutation on GJ protein levels (ogre) and GJ assembly (width of the GJ belt) (Fig. 7P-7Q).

8. ***Finally, may I suggest a reorganization of Fig 1. The initial introduction of dendrite intercalation around apodemes is quite nice, but most of phenotypic analysis of mir-14 mutant in this manuscript is about the dendrite orientation defects in other epidermal cells. Also, mir-14 mutants present multiple defects in addition to increased dendrite intercalation. It would be nice the manuscript starting with genetic screens for Dcr1 and miR14. Then the intercalation phenotype could be introduced for further study and suggest they are similar to dendrites near apodemes.***
We agree with the Reviewer that the proposed structure would likely be effective. However, we intentionally chose to introduce apodemes early in Figure 1 as (1) we felt that the overview of dendrite distribution in each segment provided an opportunity to simultaneously view the two dendrite arrangements in the same specimens and (2) our motivation in studying *Dcr* was that the phenotype suggested it may be an entry point to understanding the basis for these two distinct arrangements. We considered omitting the studies of *Dcr* altogether to simplify the narrative, however we believe that having mutations in an additional gene in the pathway yielding the same phenotypes (intercalation, nociceptive sensitization) provides further support for our findings overall.

Reviewer 2:

The Drosophila larva detects nociceptive stimuli in the environment using class IV da neurons that have free dendrite endings. Although a lot has been known about the sensory functions and dendrite organization of these neurons on the body wall epidermis, how dendrite-epidermal cell spatial distribution and neuronal sensitivity are linked was previously not very well understood. In this manuscript by Luedke at al., the authors identified an interesting mechanism by which the intercalation of sensory dendrites in the epidermal cell lateral junction is regulated by miR-14. They found that miR-14 is expressed in higher levels in regular epidermal cells but is down regulated in epodemes, so that junction intercalation of dendrites is only present at epodemes. They further found that miR-14 does so by regulating gap junction proteins, including Ogre and Inx2, expressed by epidermal cells. Lastly, they found that junction intercalation of dendrites sensitizes neurons in ways that are independent of other known pathways of sensitization.

Overall, this is a very nice study presenting novel and interesting mechanisms of dendrite/epidermis interaction and neuronal function. The investigations are multilayered and are complementary with one another. The data presented are compressive and of high quality. The evidence for their main claims is generally compelling. I support acceptance of this paper after minor revisions through additional control experiments and text editing.

<u>Major concerns:</u>

1. ***In Figure 5, the authors showed the effects of removing various channels in the miR-14Δ1 background on mechanonociceptive response of larvae. These channel mutants should be examined in the wildtype background as well to serve as controls.***
We thank the Reviewer for alerting us to this omission. Concurrent with the assays presented in the original submission we performed assays on the channel mutants, and we include those results in the revised manuscript (Fig. 5S1F).

2. ***Does integrin overexpression in miR-14 mutant cause dendrite reduction (Figure 6D)? If so, the reduced sensitivity could also be due to dendrite reduction?***
The Reviewer raises an important point that we overlooked in our original submission. To evaluate whether integrin overexpression affects dendrite length in *miR-14* mutants, we measured dendrite length and terminal dendrite number (normalized to the field size sampled) from *miR-14* controls and *miR-14* overexpressing integrins and observed no significant difference in either metric. We incorporated these new results into Fig. 6F.

3. ***The authors claim that miR-14 regulates the expression and distribution of Ogre and Inx2, however, the only results presented are representative images in Figure 7L-O. To make such claims, quantitative analyses are necessary*.**
We appreciate the Reviewer drawing our attention to this omission. In the revised manuscript we have incorporated additional analyses (ogre staining intensity normalized to armadillo staining intensity; mean width of GJ belt) and clarified our wording in the text. The new results are presented as Figure 7P-7Q.

Minor concerns:

1. Is there anything else known about mda-1? Which gene does it encode? What kind of molecule is it? The information should be included if available.

We agree that this is an intriguing allele. We sequenced the allele along with other mutants from the same screen and an isogenic control and have not found unique "highly mutagenic" polymorphisms associated with this allele. Hence, the lesion likely maps to a non-coding exon or intergenic region, but defining such a lesion will require recombination mapping and resequencing, which is likely to take a substantial amount of time and effort. In the revision, we focused the discussion of *mda1* onto the salient features that we're hoping to convey: *mda1* enhances the extent of junctional alignment in *miR-14* mutants and additionally enhances the nociceptive sensitivity, further defining the dynamic range of the system.

2. The authors could consider including a little more context about the RNA-seq experiment. Currently it is only very briefly mentioned.

We appreciate the suggestion. In the revised manuscript, we provide a more thorough explanation of our approach and rationale behind the experiments. We additionally incorporated new RNA-seq results: comparison of the transcriptomes of apodemes and epidermal cells, differential expression analysis of epidermal cell transcriptomes from control and *miR-14* mutant larvae, and the intersection of the two datasets (ie, how many of the transcripts that are differentially expressed in apodemes are deregulated in *miR-14* mutants). The new results demonstrate that neither *Inx* genes, other junction components, or other core AJ/SJ genes are likely direct targets of *miR-14*, as none of these genes are significantly deregulated in *miR-14* mutant epidermal cells.

3. Figure 8: Are groups mislabeled or bars mis-color-coded in 8M? How is apodemes labeled in K-L'? Does Tig LOF cause changes in other parts of the neuron?

The groups/bars were not mislabeled, but the color scheme (bars outlined) made the panel unnecessarily complex. We've simplified the color scheme and increased the spacing between the two groups (aligned and invading) to clarify the plot.

Apodemes are labeled by NrxIV::GFP; as described in the methods, an apodeme mask (which is shown) was generated using the NrxIV::GFP signal and dendrite alignment/invasion was assessed using that mask.

The Reviewer asks an important question that was overlooked in the prior submission: whether *Tig* LOF alters dendrite morphogenesis outside the apodeme domain. To evaluate this possibility, we measured total dendrite length outside of apodeme domains in control and *Tig* LOF mutant larvae and observed no significant difference. These new results are incorporated into Figure 8 (Fig. 8K).

4. *miR-14 is known to suppress cell death and to regulate fat metabolism and insulin production. The authors may want to discuss other possible ways in which miR-14 indirectly affects neuronal activity.*

We appreciate this suggestion. We explored possible connections to several previously defined *miR-14* targets including the cell death pathway, insulin signaling pathway, and IP3 signaling. However, we found no compelling evidence for any of these pathways in control of epidermal dendrite alignment or nociceptive sensitization. We briefly allude to these studies in the discussion.

Reviewer 3

Luedke et al present a fascinating study of phenotypes that are observed in Drosophila mutants for Dicer-1 and mir-14. Mutations in both genes are found to affect the dendritic arborization o fnociceptive neurons in the Drosophila larvae. The sensory dendrites of mutants for these genes are found to penetrate through the cell-cell boundaries of overlying epidermal cells, a pattern that is not normally seen in wild type larvae. Multiple and convincing lines of evidence suggest that mir-14 is required in epidermal cells to prevent the dendrites from penetrating at these boundaries. Furthermore, it is found that the mir-14 mutants have hypersensitive mechanical nociception and this phenotype is correlated with the boundary penetration of the dendrites as it can be suppressed by overexpression of integrin genes. Finally, it is found that epidermal gap junctions are disrupted by mir-14 mutations and manipulating gap junction gene expression in epidermal cells phenocopies the dendrite and behavioral effects. Overall, this is a very exciting study that would be of great interest to the readers of PLoS Genetics but there are some questions that have been raised by the findings and their interpretation that should be addressed.

<u>Major Points:</u>
1.) ***The authors dedicate a lot of discussion to the distinction between apodemes (aka muscle attachment sites) and epidermal cells. It is nice that this paper describes the observation that the dendrites of nociceptive neurons pass between the muscles at the site of their attachment as this is something that is well-known to those of us that are interested in these neurons, but this has not been well-described in the literature. However, throughout the manuscript the reader is given the impression that there is something special about the apodemes themselves that causes the dendrites to pass around their boundaries. There is no discussion whatsoever of the fact that the muscles, which attach to these apodemes, present a physical barrier that the dendrites simply cannot pass through. While it is possible that there are cues that guide the dendrites along the apodemal boundaries, it seems more plausible that the muscles themselves are the factor that block the dendrites from entering into this territory. This latter idea is actually supported by the authors own exciting data which are presented in figure 8L! Tiggrin mutants, which are defective in muscle attachment, show dendrite penetration into the epidermal territory. Can the authors revise the manuscript to more clearly present the tissue level structure at the apodemes? There's a muscle in the way.***

We appreciate the Reviewer's perspective and agree that a more complete discussion of the anatomy at apodemes is warranted. We included a brief discussion of muscle attachment at apodemes in our anatomical overview:

> *Whether these distinct orientations reflect physical occlusion of apodeme territory by muscles, the presence of attractive cues at apodeme junctional domains, and/or the repulsive cues at other epidermal cell-cell interfaces is currently unknown.*

In addition, we added new imaging data documenting the relative arrangement of muscle adhesion sites and dendrites of nociceptive neurons (Fig. 8G), as well as a brief discussion of the likely contribution of muscle adhesions to dendrite orientation over apodemes. Although a detailed study of muscle cues that influence dendrite morphology merits its own independent study, we believe that these images provide a fuller picture of the cell-cell interactions that likely contribute to dendrite positioning at apodemes.

2.) *Although the Tiggrin phenotype is suggestive that muscles pose a physical barrier to dendrites, it is also possible that the edges of apodemes provide some additional guidance cues to facilitate the dendrites passing around their edges. And it seems like the authors have found that low expression of gap junctions could be the relevant cues. Does mir-14 expression in epidermal cells suppress the apodeme fate? It seems like it would be interesting/important to investigate whether or not epidermal cells in mir-14 mutants show expression of other apodemal markers, there are good markers out there to look at this quickly and with little effort.*

Embedded in the comment are two important points. First, based on the dendrite distribution in controls and *Tig* mutants the Reviewer infers that apodemes likely serve as the source of guidance cue(s) which contribute to dendrite positioning. We made a similar inference, and from our RNA-seq studies have identified an apodeme-derived guidance cue. While the detailed discussion of the cue and the experimental evidence to support its function in this context is beyond the scope of the current study, we agree that a more complete discussion of the *Tig* mutant phenotype is warranted. We therefore added a sentence to the results discussing the effects of *Tig* mutants on dendrite positioning and the possibility that it might reflect a function for apodemes in providing guidance cues. Second, the Reviewer raises the incisive question of whether *miR-14* suppresses apodeme fate, which was our initial working model. To address this possibility, we took three experimental approaches:

(a) We assayed effects of epidermal knockdown of apodeme fate genes (*sr, Tsp, Taxi, Tig*) on suppressing *miR-14* dendrite intercalation phenotypes. Although we observed no effect of knockdown of any of these genes, we omitted them from the manuscript because we could not be certain about the efficacy of knockdown.

(b) We took a more unbiased approach to address the question: we used RNA-seq to identify transcriptional differences between apodemes and other epidermal cells and

queried whether apodeme-enriched (or depleted) transcripts were deregulated in epidermal cells of *miR-14* mutants. Notably, we observed negligible overlap in apodeme marker genes and epidermal *miR-14*-responsive transcripts. We include these new results in the manuscript as Figure 4S2A-4S2B.

(c) We assayed the ability of *miR-14* overexpression in apodemes to influence dendrite patterning in apodeme domains. We found that *miR-14* overexpression triggered increased dendrite invasion into apodeme domains (albeit less extensively than *Tig* LOF), but the overall organization of dendrites around apodemes as well as the general organization/positioning of apodemes was comparable to controls. Taken together with our RNA-seq studies, we conclude that *miR-14* is unlikely a suppressor of apodeme fate; instead, we propose that miR-14 regulates cell-cell interactions in the epidermis that gate the accessibility of junctional domains to dendrites. At apodemes, exclusionary interactions between dendrites and muscle adhesions likely contribute to the extensive intercalation. We updated our discussion to more clearly present this model.

3.) ***While it seems very clear that the mir-14 mutants show hypersensitive mechanical nociception, it is less clear that the penetration of dendrites into epidermal junctions has anything to do with wild type nociception. This statement does is not meant to detract from the interesting phenotype, but it is not clear that wild type neurons penetrate at these boundaries at all (besides at apodemes). Is the fraction of dendrites aligned with epidermal cells in Figure 1K significantly above the alignment that would be expected by chance? If not, the manuscript should be revised to state that normal nociception may or may not be influenced by these specific dendrite epidermal interactions.***
We agree that in the absence of clear experimental evidence that the baseline level of epidermal intercalation influences nociception, the text should be modified. We modified the discussion (final paragraph and pasted below) according to the suggestion.

> *In miR-14 mutants, C4da dendrites inappropriately intercalate between epidermal cells outside of apodeme cells, and our studies suggest that this increased dendrite-epidermis coupling enhances nociceptive sensitivity. It remains to be determined whether the low level of intercalation that normally occurs outside of apodeme domains (~4% of the arbor; Fig. 1G) contributes to nociceptive responses in wild-type larvae.*

4.) ***The data presented in figure 8 G-J are completely unconvincing. The signals in panel I do not resemble genuine GCaMP responses. The description of the methodology employed is not adequate for these data to be evaluated by a reviewer. These data add very little information to the manuscript, and the easiest thing for the authors to do would be to just remove the data. It is extremely challenging to rigorously analyze calcium responses in a moving preparation and these experiments are not credible as currently presented.***
The Reviewer presents a valid critique of the experiments; we appreciate the frank assessment. We unsuccessfully attempted to repeat the experiments with ratiometric calcium reporters and therefore removed the imaging from the revised manuscript.

5.) ***It's interesting that mir-14 mutants don't cause thermal hyperalgesia and the effect is specific to mechanical, recommend moving Figure 5D supplement 1D to main figure.***
We agree with the Reviewer's assessment of the result; this was one of the early results that truly focused our interest on *miR-14*. Rather than moving the results from 5S1D which assess thermal allodynia, we incorporated additional results into Figure 5 depicting *miR-14* mutant behavioral responses to a range of thermal inputs.

6.) ***Congratulations to the authors for a beautiful and fascinating study.***
We appreciate the Reviewer's constructive critiques!
